# Neuroinflammation mechanisms of neuromodulation therapies for anxiety and depression

**DOI:** 10.1038/s41398-022-02297-y

**Published:** 2023-01-09

**Authors:** Bingqi Guo, Mengyao Zhang, Wensi Hao, Yuping Wang, Tingting Zhang, Chunyan Liu

**Affiliations:** 1 Department of Neurology, Xuanwu Hospital, Capital Medical University, Beijing, 100053 China; 2 Beijing Key Laboratory of Neuromodulation, Beijing, 100053 China; 3 Institute of sleep and consciousness disorders, Center of Epilepsy, Beijing Institute for Brain Disorders, Capital Medical University, Beijing, 100069 China

**Keywords:** Depression, Physiology

## Abstract

Mood disorders are associated with elevated inflammation, and the reduction of symptoms after multiple treatments is often accompanied by pro-inflammation restoration. A variety of neuromodulation techniques that regulate regional brain activities have been used to treat refractory mood disorders. However, their efficacy varies from person to person and lack reliable indicator. This review summarizes clinical and animal studies on inflammation in neural circuits related to anxiety and depression and the evidence that neuromodulation therapies regulate neuroinflammation in the treatment of neurological diseases. Neuromodulation therapies, including transcranial magnetic stimulation (TMS), transcranial electrical stimulation (TES), electroconvulsive therapy (ECT), photobiomodulation (PBM), transcranial ultrasound stimulation (TUS), deep brain stimulation (DBS), and vagus nerve stimulation (VNS), all have been reported to attenuate neuroinflammation and reduce the release of pro-inflammatory factors, which may be one of the reasons for mood improvement. This review provides a better understanding of the effective mechanism of neuromodulation therapies and indicates that inflammatory biomarkers may serve as a reference for the assessment of pathological conditions and treatment options in anxiety and depression.

## Introduction

The prevalence of anxiety and depression continues to increase. Anxiety disorders include generalized anxiety disorder, panic disorder, social anxiety disorder, agoraphobia, and specific phobias, affecting 374 million people worldwide (4802 cases per 100,000 people) [1]. Depression, characterized by low mood, slowed thinking, and reduced volitional activity, affects 221 million people worldwide (3,152.9 cases per 100,000 people) in 2020 [1]. The Lancet estimated that the coronavirus disease 2019 (COVID-19) pandemic has resulted in an additional 53.2 million cases of major depressive disorder, which led to an increase in total disability-adjusted life years (DALYs) to 49.4 million, and 76.2 million cases of anxiety disorders, with total DALYs to 44.5 million worldwide in 2020 [1]. Symptoms of anxiety and depression often coexist, and comorbidity is associated with more severe symptoms, worse quality of life, greater recurrence, and higher suicide risk than either disorder alone. Despite the increasing incidence rates and heavy burden, the treatment effect for anxiety and depression is unsatisfactory. Conventional first-line treatment, including psychotherapy and medication, only achieves a 50% remission rate [2].

Neuromodulation techniques, including TMS, TES, ECT, DBS, and VNS, as well as the promising transcranial PBM and TUS, provide important adjunctive therapies for the treatment of anxiety and depression disorders [3, 4]. The therapeutic power of neuromodulation comes from its ability to modulate the neural activity of specific brain regions and the related network function [5]. However, because of an insufficient understanding of the etiopathogenesis and pathophysiology of psychiatric disorders, even for a given symptom, the effective targets may vary from patient to patient. In addition, treatment results are generally determined by the patient’s report of symptoms, which may be lagging and unreliable. Thus, it is critical to identify objective markers to guide the formulation of treatment plans and evaluate their effectiveness.

**Fig. 1:**
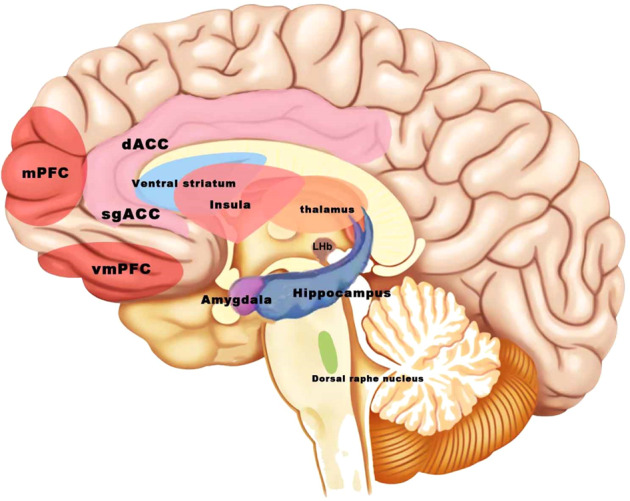
Brain regions with inflammatory activation in the key circuits of anxiety and depression. Altered inflammation in the medial prefrontal cortex (mPFC), anterior cingulate cortex (ACC), amygdala, hippocampus, insula, and other brain areas has been reported in vivo PET imaging studies in humans. The lateral habenula (LHb) and the dorsal raphe nuclei (DRN), which play an important role in depression, are found to have inflammatory changes in animal studies.

Numerous studies have confirmed the association between chronic inflammation and depression, anxiety, and other psychiatric disorders, particularly those refractory to conventional medications [6–8]. Elevated levels of peripheral inflammatory biomarkers are present in patients with depression, and the level of inflammation correlates with the severity of specific symptoms [9]. Studies using positron emission computed tomography (PET) imaging with the 18 kDa translocator protein (TSPO) as a biomarker of microglia have shown that neuroinflammation exists in multiple brain regions in depression patients [10–15] (Fig. 1). Studies using animal models have also revealed pro-inflammatory factor release and microglial activation in the brains of animals, showing signs of anxiety and depression [16–19]. Interventions using drug therapy and neuromodulation for psychiatric disorders have been shown to reduce inflammation while relieving symptoms [18, 20–27]. We hypothesized that changes in inflammatory indices could be effective indicators for neuromodulation therapy. This review examines inflammatory alterations in the brain regions involved in anxiety-related threat and fear circuits and depression-related reward circuits. Furthermore, the modulation of inflammation in these key brain areas by various neuromodulation therapies was explored, with the aim of providing a reference for basic and applied research on neuromodulation therapy for the treatment of psychiatric disorders such as anxiety and depression.

## Key neural circuits and the brain regions involved in anxiety and depression disorders

Decades of studies have identified the brain areas involved in the pathophysiology of anxiety and depression. Owing to the development of optogenetic and chemogenetic techniques, neural circuits can be studied at higher spatial rates through finer control of neuronal activity, and the functional complexity of brain regions and nuclei has been discovered in animals. For example, BLA projections to the PFC, hippocampus, and lateral central amygdala (CeL) have a pro-anxiety effect, while projections to the bed nucleus of the terminal striatum and medial central amygdala (CeM) have an anti-anxiety effect. Activation of the medial prefrontal cortex (mPFC)-dorsal raphe nucleus (DRN) has an antidepressant effect, while activation of the mPFC-lateral habenula (LHb) has a pro-depressant effect [28]. The subcortical projection subgroup of the DRN (represented by the projection to the CeA) promotes anxiety, and the cortical projection subgroup (represented by the projection to the prefrontal cortex) is related to reward function [29]. In parallel, studies in humans revealed the role of large-scale functional brain networks in depression and anxiety. For example, major depressive disorder (MDD) was characterized by hyperconnectivity between default network seeds and regions of the hippocampus extending to the middle temporal gyrus, and areas of mPFC. These areas are believed to support internal mentation, e.g., self-referential thinking and affective decision-making [30, 31].

## Inflammatory changes in the specific brain regions of threat and fear circuits

Classically, the neural circuits closely associated with anxiety disorders are the threat-fear circuit [32], which includes the dorsolateral prefrontal cortex (DLPFC, not present in rodents), medial prefrontal cortex (mPFC, which corresponds to the medial precentral area, anterior cingulate cortex (ACC), prelimbic cortex (PL), and infralimbic cortex (IL) in rodents, with IL appearing to correspond to the subgenual cortex area, for example, [Brodmann area 25]) [33], amygdala (including the basolateral amygdala (BLA), lateral amygdala (LA), central amygdala (CeA), terminal bed nucleus), hippocampus, anterior insula, and other brain regions and nuclei. In general, external stimuli are transmitted to the amygdala and terminal bed nucleus through the thalamus and cortex. As the center of fear, the amygdala changes behaviors (freezing and startle), activate the autonomic nervous system, and transmit information to the hippocampus and bed nucleus of the terminal striatum [34]. PFC uses information gathered from various cortical and subcortical processing streams to predict the likelihood of threats in the environment, however, a persistent bias towards threat prediction can lead to a state of over-engagement in the defense system and anxiety [35]. Therefore, connections between these subcortical regions and the PFC play an important role in regulating anxiety. Altered inflammation in the amygdala, medial prefrontal cortex, dorsal cingulate, hippocampus, insula, and other brain areas has been reported in animal models and clinical studies.

### Amygdala

The amygdala is a key brain region in emotion regulation and the generation of fear and anxiety. In recent years, studies have shown that social pressure and stress can cause inflammation, leading to changes in the human amygdala and related neural circuits, while changes in the amygdala activity can worsen inflammation, forming positive feedback. As early as 1982, Henke et al. found that stimulation of the CeA and anterior cingulate had a pro-inflammatory effect on gastric ulcers and that damage to these sites prevented stress from exacerbating gastric inflammation. This suggests that amygdala activity may have a pro-inflammatory effect and is not manifested only in the brain [36]. Conversely, inflammation increases amygdala activity [37], and individuals with higher levels of inflammation have a more active amygdala in response to social threats [38]. Acute social stress from an interview showed that increased amygdala activity and its strong coupling with the DLPFC were associated with higher levels of inflammation (higher interleukin or IL-6 and tumor necrosis factor or TNF-α) [39]. Zheng et al. found that microglial activation and pro-inflammatory cytokine production in the lateral amygdala and increased presynaptic glutamate release in a mouse model of lipopolysaccharide-induced neuroinflammation resulted in excitatory/inhibitory (E/I) imbalance, and that mice exhibited anxiety and depression-like behavior [40]. The anti-inflammatory factor IL-10 reverses abnormal gamma-aminobutyric acid (GABA) transmission in the amygdala, anxiety-like behavior, and substance dependence [41]. In adult male Sprague-Dawley rats, repeated social defeat leads to increased activation of microglia in the BLA and increased BLA discharge, whereas blocking microglial activation prevents anxiety-like behavior [16]. This indicates that there may be a mutually reinforcing effect between amygdala activity and inflammation levels, ultimately leading to anxiety.

### Medial prefrontal and dorsal anterior cingulate cortices

Regions of the mPFC, including the rostral ACC, sgACC (Brodmann area 25), and medial frontal gyrus, have extensive relationships between the amygdala and others and are thought to be involved in emotion regulation. The dACC, often activated in patients with anxiety disorders, plays an important role in error detection, conflict monitoring [42], dealing with socially distressing emotions, and top-down activation of the autonomic nervous system [43].

Numerous neuroimaging studies have shown that elevated pro-inflammatory factors alter the activation of the mPFC and dACC. Women experiencing bereavement have increased IL-1β and TNF receptor II levels in their blood and show activation of the ventral mPFC (including the sgACC and orbitofrontal cortex) [44]. Healthy subjects have increased blood IL-6 levels, activation of the sgACC and dACC, and decreased functional connectivity between the sgACC, amygdala, and mPFC, as well as depressed mood after typhoid vaccination [45, 46]. Increased activation of the dACC is found in subjects with increased neuroticism and obsessive-compulsive disorder, both of which are associated with increased anxiety and arousal, as well as increased inflammatory markers [47]. A neuroimaging study in patients with hepatitis C showed that treatment with interferon-alpha led to increased dACC activity [48]. Cytokine stimulation (Interferon or IFN-α) for 12 weeks in patients who are HCV-positive resulted in an increase in dACC activity in response to visuospatial attention error monitoring [48]. The above results suggest that neural activity in the mPFC and dACC is closely related to inflammation. In animal studies, the activation of the microglia and the elevation of the pro-inflammatory factors IL-1α and TNF-α were evident in the mPFC of mice with anxiety induced by repeated social deficits. Chronic stress has long been found to cause alterations in the neuronal function of the mPFC in humans and animals and Hinwood et al. found that such alterations may be related to the activation of the mPFC microglia [49]. Administration of complete Freud’s adjuvant (CFA) to mice-induced pain and anxiety-like behavior, significantly increased the expression of p-P38 and p-JNK in the ACC (this signaling pathway functions as a cytokine-inducing activator), activated the microglia and astrocytes, and increased the levels of pro-inflammatory factors IL-1β, TNF-α, and IL-6 [50].

### Hippocampus

The hippocampus is located between the thalamus and medial temporal lobe of the brain and is part of the limbic system, involved in the regulation of cognitive functions, and has extensive neural network connections with emotion-related brain areas (the prefrontal cortex and amygdala). Several magnetic resonance imaging (MRI) studies have shown that amygdala and hippocampus volumes are significantly smaller in patients with post-traumatic stress disorder (PTSD) and social phobia than in healthy controls [51, 52]. Numerous animal studies have shown that chronic stress leads to elevated levels of pro-inflammatory factors in the hippocampus and activation of the NLR family pyrin domain containing 3 (NLRP3) inflammatory vesicles, a component of the innate immune system that acts as a pattern recognition receptor (PRR) and recognizes pathogen-associated molecular patterns (PAMP). The NLRP3 forms a caspase-1 activation complex, namely, NLRP3 inflammasomes, with adapter ASC protein PYCARD to shear the precursor of cytokine IL-1β and release active IL-1β [53]. TNF-α induced by the hippocampal microglia is significantly elevated in mice exposed to acute stress [54], and cytokines (IL-1β, TNF-α) released by the microglia can inhibit neurogenesis in the dentate gyrus [55], thereby promoting neuronal apoptosis [56] and increasing anxiety-related behavior [57]. In a meta-analysis encompassing multiple animal models of stress, all studies showed increased expression of the microglial cell surface marker ionized calcium-binding adapter molecule 1 (Iba1) in the hippocampus, and 75% of the studies showed increased Iba1 in the prefrontal cortex [58]. The hippocampal dentate gyrus of highly trait-anxious mice exhibited enhanced Iba1+ density and CD68+/Iba1+ microglial density. Oral administration of the microglial inhibitor, minocycline, reduced these changes and alleviated hyper anxiety in mice [23]. In a mouse model of PTSD constructed by footshock and situational reminder, the number of microglia in the hippocampus, prefrontal cortex, and amygdala was significantly increased and activated [59]. Twelve weeks of chronic mild stress-induced anxiety-like behavior in rats, and hippocampal microglial activation was detected in vivo by (18F) DPA-714 PET and in vitro by immunofluorescence staining and protein blotting [18].

### Insula

In a meta-analysis of functional magnetic resonance imaging (fMRI) and PET in patients with PTSD, social anxiety disorder, atopic phobia, and in healthy individuals in fearful situations, patients showed stronger activity in the amygdala and insula than the controls did [60]. In healthy subjects injected with endotoxin, increased levels of glucose metabolism in the insula and decreased levels of metabolism in the ACC were observed using PET [61], decreased resting-state functional connectivity between the amygdala, anterior insula, and cingulate cortex was observed using fMRI [62]. In addition, endotoxin selectively enhanced amygdala activity while subjects were assessing socially threatening images [63], interestingly, female subjects (but not males) had increased activity in the dACC and anterior insula with increased IL-6 in response to social exclusion [64].

The above studies have shown that inflammation can affect the activity of anxiety-related brain regions such as the amygdala, ACC, insula, and functional connections within the circuit, thus causing emotional problems such as anxiety and reducing social adaptation.

## Inflammatory changes in the specific brain regions of associated reward circuit in depression

The neural circuits closely associated with depression are the reward circuit and aversion center, mainly include the ACC, ventral tegmental area (VTA), ventral striatum comprising the nucleus accumbens (NAc) and ventral pallidum (VP), raphe nucleus, orbital prefrontal cortex (OFC) [65]. The LHb is the aversive center which produces negative emotions when active. Recent studies have found that ketamine targeting LHb has rapid and effective antidepressant effects [66]. In recent years, studies have strongly confirmed the presence of neuroinflammation as manifested by microglial activation in human emotional disorders, and this change varies with the course of the disease and treatment [10, 11]. In a subsequent study, the authors confirmed that the duration of untreated major depression was a strong predictor of TSPO distribution volume (VT), that microglial activation was higher in depressive patients who had not been treated with medication for a longer time than in those with a shorter course of the disease, and that the degree of microglial activation no longer increased yearly when antidepressants were administered [13, 14].

### Prefrontal cortex and anterior cingulate cortex

In autopsies of suicidal patients with depression, the total microglia density was found to be no different from controls in the dACC. However, the proportion of primed over the ramified microglia was elevated, and the primed microglia expressed MHC class II antigen and CD68, leading to persistent neuroinflammation affecting neuronal function and causing psychiatric disorders. The presence of large numbers of macrophages in the perivascular area and the increased expression of Iba1 and monocyte chemoattractant protein-1 (MCP-1) suggest that the peripheral mononuclear cells were recruited by microglia and converted into macrophages in this brain region to participate in neuroinflammation [67, 68]. Setiawan et al. performed PET with [18F] FEPPA in 20 patients with major depression and 20 healthy controls. The results showed that TSPO VT was significantly higher in the prefrontal cortex, ACC, and insula suggesting microglial activation, with TSPO VT in the ACC correlating with the severity of depression [15]. After typhoid vaccination in healthy individuals, the activity of the sgACC (associated with the etiology of depression) was enhanced in response to a task that implied an emotional face. In addition, inflammation reduces the connectivity of the brain regions involved in emotional processing, such as the sgACC to the amygdala, medial prefrontal cortex, nucleus accumbens, and superior temporal sulcus [45].

Animal models have demonstrated that chronic unpredictable mild stress(CUMS) promotes the production of pro-inflammatory cytokines in the mPFC. In the prefrontal area of stress-susceptible mice, the expression of TNF-α, cyclooxygenase (COX)-1, and Iba-1positive microglia cells increased [69]. Pan et al. found that a 12-week CUMS procedure remarkably increased PFC IL-1β mRNA and protein levels in depressive-like behavior of rats, and induced the activation of NLRP3 inflammasome. Moreover, the increased co-location of NLRP3 and Iba1 protein expression supported that microglia was the primary contributor to CUMS-induced neuroinflammation [70].

### Ventral striatum

The ventral striatum, part of the striatum, is connected to the limbic system and receives nerve fiber projections from the prefrontal cortex (including OFC, vmPFC, and dACC), hippocampus, amygdala, and dopamine neurons in the VTA of the midbrain. It is involved in reward and emotional responses and is thought to be central to the brain’s reward system.

High levels of inflammation in patients with depression have been shown to affect cortical striatal reward circuits. In a resting-state fMRI of unmedicated patients with major depression, increased C-reactive protein (CRP) was associated with reduced connectivity between the ventral striatum and vmPFC and that of the dorsal striatum with the vmPFC and supplementary motor area (SMA), which are associated with depression’s core symptoms of bradykinesia and psychomotor slowing, respectively [71]. Yin et al. collected fMRI data from depressive patients with different levels of inflammation and found that increased plasma CRP was associated with reduced connectivity in widely distributed networks in the ventral striatum, ventral medial prefrontal lobe, and amygdala and that feeding these multivariate network features into machine learning algorithms could predict depressive symptoms with high accuracy [72]. In addition, artificially high levels of inflammation not only cause depressive mood but also alter ventral striatal activity and behavior responses to rewards and punishments [73, 74]. Compared with placebo, healthy subjects injected with low doses of endotoxin experienced more pronounced depression and significantly reduced neural activity in the ventral striatum when participating in a task that was expected to receive a monetary reward [75]. When healthy volunteers who were vaccinated against typhoid faced the task of choosing a high-probability reward (win £1) and avoiding a high-probability punishment (loss £1), inflammation caused the ventral striatum and insula to mispredict reward and punishment, making the potential reward less attractive and the punishment more distasteful [73]. In healthy women, after experiencing the Maastricht Acute Stress Test and Montreal Imaging Stress Task, when completing a reward-punishment probability task paradigm, the ventral striatal reward prediction error signal transmission was decreased and correlated with an increase in IL-6 [74].

### Dorsal raphe nucleus

The dorsal raphe nuclei (DRN) is located in a narrow area near the median suture of the brainstem, which is the largest septum nucleus and predominant 5-hydroxytryptamine neuron nucleus in the central nervous system. It is thought to be closely associated with psychiatric disorders such as anhedonia, anxiety, and depression. After stimulation by inflammatory factors, such as IL-1β, LPS, TNF-α, and Aβ42, microglia in the DRN are activated, IDO expression in the neurons is increased, the expression of tryptophan-5-hydroxylase (TPH, the rate-limiting enzyme in 5-hydroxytryptamine synthesis) is decreased, and the nucleus swells and degenerates [76]. Patients with inflammatory bowel diseases are often associated with psychiatric disorders such as depression or anxiety. Dextran sulfate sodium (DSS)-induced colitis rats exhibited depressive-like behavior and increased expression of the immediate-early gene FosB/ΔFosB, inducible nitric oxide synthase (iNOS), and reactive microglia in the DRN during the resolution phase of experimental colitis. Persistent central inflammation, particularly that of the DRN, may play an important role in the progression of depression and anxiety [77].

### Lateral habenula

The habenula is located in the posterior part of the thalamus near the midline and can be divided into two regions: the medial habenula (MHb) and LHb. The LHb receives afferent information mainly from the basal ganglia and limbic forebrain and projects mainly to the rostromedial tegmental nucleus (RMTg) and midbrain monoaminergic nuclei. In various animal models of depression, the LHb is the only brain region that shows consistently increased activity, and a large body of evidence from animal models and human studies suggests a relationship between the LHb and various psychiatric disorders, particularly major depression [78].

Chronic unpredictable stress (CUS) increases the nuclear factor-κB (NF-kB) signaling pathway expression in the LHb, and injection of TNF-α into the LHb leads to depressive-like behavior in rats, which is conversely reduced by anti-inflammatory aspirin or NF-kB inhibitors [79]. Destruction of the LHb reduces inflammatory responses in the hippocampus and ameliorates hippocampal degeneration by reducing the expression of the PI3K/mTOR signaling pathway and apoptosis-related proteins, including phosphorylated p53, Bax, Bcl-2, and cleaved-caspase3, demonstrating the role of inflammatory responses of the LHb in depression [79]. Chronic social defeat stress (CSDS) causes depression in rodents, and RNA-seq analysis shows that this is associated with promoting the production of proprotein convertase subtilisin/kexin type 5 (Pcsk5) in LHb neurons, which activates the matrix metalloproteinase (MMP) 14-MMP2 pathway and promotes remodeling of the extracellular matrix to produce neuroinflammation [80].

## Neuromodulation therapy reduces cytokines production and improves microglial function

Physical neuromodulation is a new therapeutic technique that has developed rapidly in recent years and includes non-invasive TMS, TES, ECT, PBM, and TUS, as well as invasive DBS and VNS. A growing body of studies has confirmed that these neuromodulation techniques can modulate inflammation while reducing psychiatric symptoms (Table 1).

**Table 1:** In vivo studies on neuromodulation therapy regulate inflammation.

Type of Neuromodulation	Disease/model	Treatment targets	Effects on inflammation	references
TMS	Patients with refractory depression	Left dorsolateral prefrontal cortex	Decrease serum IL-1β and TNF-α	Zhao et al. [87]
	MDD patients	Bilateral DLPFC	No significant difference	Chou et al. [88]
	CUMS rat model	Vertex of the skull	Decrease hippocampus TNF-α, iNOS, IL-1β, and IL-6	Tian et al. [24]
TES	Bipolar depression patients MDD patients AD patients	DLPFC Anodal on left DLPFC and Cathodal on right DLPFC Bitemporal lobes(40 Hz tACS)	Decrease plasma IL-6 and IL-8 No significant difference Decrease of microglia activation	Goerigk et al. [21] Brunoni et al. (2014), Brunoni et al. [98] Dhaynaut et al. [100]
	Rats model of vascular dementia Healthy mice MCAO mouse model	2.5 mm posterior to bregma AP + 0.5 mm and ML + 1.5 mm from bregma(anodal tDCS) Ischemic hemispheres	Decrease hippocampal IL-1β, IL-6, and TNF-α reduce Iba1-positive microglia Increase the number of iNOS-positive M1-polarized microglia	Guo et al. [94] Pikhovych et al. [95] Braun et al. [96]
ECT	Resistant major depression patients	Right unilateral and bilateral ECT	Activate peripheral blood mononuclear cells, increases circulating IL-1β, IL-6	Fluitman et al. [108], Lehtimäki et al. [109]
	CUMS rat model	Bilateral ear clip	Increase hippocampal IL-1β and TNF-α	Zhu et al. (2015)
	Depression patients	Bitemporal	Decrease in IL-6 levels	Belge et al. (2020)
	Autoimmune encephalomyelitis	Ear clip	Reduce microglia activation, T cell stimulatory and chemokine expression	Goldfarb et al. [105]
**Photobiomodulation**	Aged rats	Cerebral cortex	Increase hippocampus IL-1α and decrease IL-5 and IL-8	Cardoso et al. [117]
**TUS**	MCAO mouse model LPS-treated mice PD mouse model	Ischemic hemispheres 2 mm posterior to the bregma STN	Increase IL-10、IL-10R and M2 microglia Inhibit activation of TLR4/NF-κB inflammatory signals and reduced TNF-α, IL-1β, and IL-6 Nnormalize the expression NF-κB, TNF-α, IL-1β and COX-2 and NF-κB	Wang et al. [93] Chen et al. [122] Zhou et al. [124]
**DBS**	Rats	Infralimbic cortex	Increase expression of glial fibrillary-acidic-protein, TNF-α, and p11	Perez-Caballero et al. [135]
	Rats	Subthalamic nucleus	Increase the density of PBR	Hirshler et al. (2010)
	FPI rat model	Lateral cerebellar nucleus	Suppress expression of pro-inflammatory genes, suppress microglial, and astrocytic activation	Chan et al. [136]
	Pilocarpine-induced SE rat model	Anterior thalamic nucleus	Countered the increase in hippocampal caspase3 activity and IL-6 levels, but had no effect on TNFα	Covolan et al. (2014)
	6-hydroxydopamine injection Rat PD model	Subthalamic nucleus	Suppress microglia activation and NF-κB expression, decrease IL-1β and IL-6, increase IL-4, downregulate IL-1R, ERK, and cleaved-caspase3	Chen et al. [138]
VNS	Migraineurs	Cervical vagus nerve	Decrease serum IL-1βdecrease in IL-8	Chaudhry et al. (2019)
	LPS endotoxemia in rats	Peripheral vagus nerve	Inhibit TNF synthesis, attenuate peak serum TNF amounts	Borovikova et al. [139]
	LPS endotoxemia in mice	Vagus nerve	Reduce the central levels of IL-1β, IL-6, and TNFα, prevent LPS-induced hippocampal microglial activation	Huffman et al. (2019) Meneses et al. [140]
	POCD-aged rat model	Auricular vagus nerve	Decrease hippocampus TNF-α, IL-1β, and the expression of NF-κB suppress the elevated level of TNF-α	Cai et al. (2019)
	CUMS and CCI rat model	Transcutaneous auricular vagus nerve	Decrease plasma and multiple brain regions TNF-α	Guo et al. [94]

*CUMS* chronic unpredictable mild stress, *STN* subthalamic nucleus, *MCAO* middle cerebral artery occlusion, *PBR* peripheral benzodiazepine receptors, reflects microglia cell density, *SE* status epilepticus, *ERK* extracellular signal-regulated kinase, *LPS* lipopolysaccharide, *TBI* traumatic brain injury, *POCD* postoperative cognitive dysfunction, *CCI* chronic constriction injury of the sciatic nerve.

### Transcranial magnetic stimulation

TMS uses magnetic fields to interfere with neural activity in local superficial areas of the brain and has been approved by the US Food and Drug Administration (FDA) for the treatment of depression, addiction, and other disorders. The direct effect of TMS is to cause changes in the membrane potential of neurons at the target site and indirectly to cause other outcomes such as neurotransmitter release, improved synaptic plasticity, increased cell survival, and altered inflammatory and immune processes [81]. Low-frequency repetitive transcranial magnetic stimulation (rTMS) of cultured astrocytes reduced the expression of calcium signals and genes related to inflammatory damage pathways [82]. High-frequency (10 and 20 Hz) rTMS not only decreased the level of TNF-α but also increased the level of the anti-inflammatory factor IL-10 and significantly inhibited the classical activation and A1 marker expression of astrocytes [83, 84]. And intermittent theta-burst stimulation of rTMS can regulate microglial polarization via TLR4/NFκB/NLRP3 signaling pathway [85]. In the rat of CUMS model, rTMS treatment significantly improved anxiety and depression-like behavior and reduced the levels of inflammatory factors TNF-α, iNOS, IL-1β, and IL-6 in the hippocampus [24]. In human studies, TMS modulates the levels of inflammatory factors during disease treatment. The most common target of TMS for depression is the left DLPFC [81], and the most commonly used for anxiety-related disorders is the right DLPFC [86]. rTMS at 10 Hz to the left DLPFC can reduce the serum levels of IL-1β and TNF-α in elderly patients with refractory depression, and this change correlates with Hamilton Depression Rating Scale (HDRS) scores [87]. Another study measured inflammatory cytokines in MDD patients who received bilateral theta-burst stimulation on DLPFC and found a slight decrease in IL-6 and CRP compared with the sham group, but not a statistically significant difference [88]. These findings suggested rTMS as a non-pharmacological approach, can target anti-inflammation, and regulate microglial function in depression and anxiety disorders.

### Transcranial electrical stimulation

TES, including constant (transcranial direct current stimulation, tDCS) or alternating currents (transcranial alternating current stimulation, tACS), modifies brain function by applying weak electrical currents to the scalp. Since its introduction in the 1980s, this method has been used to improve a variety of diseases, such as mood [89], cognitive [90], and motor disorders [91]. It is generally accepted that TES can alter cortical excitability, for example, anodal stimulation has an excitatory effect, cathodal stimulation has an inhibitory effect, and alternating current can modulate endogenous brain oscillations [92, 93]. Accumulating evidence suggested that non-invasive TES could affect inflammatory response and microglial function. tDCS treatment inhibits the expression of IL-1β, IL-6, and TNF-α in rats, thereby reducing the hippocampal inflammatory response [94]. Pikhovych et al. suggested anodal tDCS to reduce Iba1-positive microglia in the cortex of healthy mouse [95]. Another experiment found that cathodal tDCS increased the number of iNOS-positive M1-polarized microglia without affecting CD 206-positive M2-polarized microglia [96]. Meanwhile, Brunoni et al. measured plasma levels of various cytokines, including IL-1β, IL-6, IL-10, and TNF-α, before and after tDCS treatment (anodal stimulation on left DLPFC and cathodal stimulation on the right DLPFC) in depressed patients, showing all of these factors decreased after tDCS treatment, but this decrease was not significantly different from the sham group [97, 98]. And using tDCS for patients with depression with type I or II bipolar disorder, not only was the patient’s higher baseline IL-6 concentration associated with therapeutic efficacy, but the patient’s plasma IL-8 concentration also decreased significantly after treatment [21]. In recent years, there has been some exploration of using tACS to treat mood disorders. Wang et al. used 77.5 Hz tACS on the forehead to treat depression and achieved positive results [99] A case series reported 40 Hz tACS leading to a significant decrease of microglia activation as measured by [11 C]-PBR28 [100]. Thus the anti-inflammatoy mechanisms of tDCS and tACS make them a particularly promising avenue in treating various emotional conditions.

### Electroconvulsive therapy

ECT is the longest-standing and most widely used neuromodulation therapy for the treatment of refractory depression, particularly for patients with suicidal tendencies or depressive symptoms. Electrodes are generally placed bilaterally in the temporal or frontal regions, and electrical stimulation is used to induce generalized seizures, thereby improving depressive symptoms. Animal studies reported a general activation of inflammatory molecules and pathways within 4 h of receiving ECT [101]. An hour after three or four ECT courses, the protein and mRNA expression of NF-κB was increased and the transcription of the COX2 gene, involved in an acute inflammatory response, was increased in rats [102, 103]. ECT also reduces microglial activation [104] and cytotoxicity by inhibiting T cell stimulatory and chemokine expression and iNOS expression, nitric oxide, and reactive oxygen species (ROS) production [105]. In human studies, the efficacy of ECT for depression are related to enhanced cortical neuroplasticity [106] and improved connectivity of the limbic system and prefrontal network [107]. And in terms of peripheral and central inflammation, some studies have suggested that ECT in patients with depression activates peripheral blood mononuclear cells in 30 min [108], increases circulating pro-inflammatory factors such as IL-1β and IL-6 at 3 h, and falls back to baseline at 24 h [109]. Conversely, other studies have suggested that ECT has anti-inflammatory effects, and researchers performing ECT in patients with depression found reduced plasma levels of pro-inflammatory factors IL-6 and TNF-α and increased hippocampal volume [110]. In addition, ECT significantly reduced plasma quinolinic acid and improved the unbalanced kynurenine pathway affecting the monoaminergic neurotransmitters in patients with major depression [111]. These findings supported the efficacy of ECT in the treatment of refractory depression potentially through a neuroinflammatory mechanism.

### Photobiomodulation

PBM is a relatively safe and well-tolerated neuromodulation technique that uses artificial light to irradiate specific brain areas. Its effectiveness in treating depression has been reported in clinical and animal models [112]. Some studies suggest a relationship between increased mitochondrial energy metabolism and local cerebral blood flow as an action mechanism of PBM [113]. In addition, light therapy may have anti-oxidative stress and anti-inflammatory effects by modulating ROS, whose brief burst can activate the redox-sensitive transcription factor, NF-kB, nitric oxide, cyclic AMP, calcium, etc. In cell experiments, PBM can reduce inflammatory markers, such as COX-2, prostaglandin E2etc, and regulate the expression and secretion of activated normal T cells [114]. Hwang et al. found that 405, 532, and 650 nm light reduced IL-8 expression, and 405 nm light also reduced IL-6 expression [115]. Huang et al. found that the antidepressant effect of PBM involved activation of the retina-ventrolateral genicular nucleus of the thalamus/interlobar-lateral habenula pathway to regulate depressive mood [116]. Laser treatment (660 nm, 100 mW) at 5 irradiation points on the head increases the level of IL-1α in the hippocampus of aged rats and decreases the levels of IL-5 and IL-8, thus improving the inflammatory response [117]. The abdomen of pregnant rats exposed to 808 nm near-infrared light was related to the effect of light on promoting the transformation of pro-inflammatory microglia to the anti-inflammatory M2 type [118]. Clinically, transcranial PBM therapy using near-infrared light in 10-Hz pulsed mode appears to be a hopeful technique for the treatment of MDD [119], suggesting that the effects of PBM on modulating neurological function in the brain for the treatment of depression may be related to the modulation of microglial activity and central inflammatory responses.

### Transcranial ultrasound stimulation

Low-intensity focused ultrasound stimulation (LIFUS) is a very promising new non-invasive neuromodulation technology. LIFUS activates or inhibits neural activity by transmitting acoustic mechanical vibrations to stimulate specific areas of the brain and has the unique advantage of high spatial specificity and targeting of deep brain nuclei compared to other neuromodulation techniques [120]. Nonthermal mechanical mechanisms such as through mechanosensitive ion channels and voltage-gated ion channels mediating altered neuronal firing [121] is the action of LIFUS for neuromodulation. Animal and cell experiments have demonstrated that LIFUS changed the expression of the inflammatory signaling pathway to play an anti-inflammatory role. LIFUS treatment was found to inhibit LPS-induced activation of TLR4/NF-κB inflammatory signals and reduced the protein levels of TNF-α, IL-1β, and IL-6 in LPS-treated mice [122]. Furthermore, LIFUS significantly decreased the Bax/Bcl-2 ratio in the microglia following LPS treatment [123]. LIFUS can normalize the expression of not only inflammatory cytokines (NF-κB, TNF-α, IL-1β) but also downstream signaling proteins such as COX-2 and NF-κB [124]. In animal studies, LIPUS stimulation of the vmPFC or left PFC improved the rat’s anxiety-depression-like behavior [125, 126]. In human studies, Sanguinetti et al. found that TUS (2 MHz, 15 s) in the right inferior frontal gyrus increased positive mood for 15–30 min in healthy subjects [127]. Reznik et al. randomly divided 24 mild-moderate depressed college students into two groups, and the true stimulation group received 5 sessions of TUS (500 kHz, PRF 40 Hz, 30 s) at the right fronto-temporal area, and the results showed that TUS improved anxiety symptoms, but not depressive symptoms. In the future, LIFUS will have a great application prospect in the treatment of neuropsychiatric diseases by influencing the inflammatory response and accurately regulating the function of a neural circuit.

### Deep brain stimulation

DBS refers to the stereotactic implantation of electrodes into specific brain regions, and the application of electrical impulses to stimulate neuronal activity for disease treatment. DBS for anxiety and depression is still in the clinical research phase and not yet mature. Some studies suggest that DBS treatment leads to neuroinflammation, including the activation of astrocytes and microglia, as confirmed in autopsies of patients treated with DBS [128–130] and in animal studies with implanted electrodes [131–133]. It has been suggested that acute inflammation caused by electrodes implantation can alleviate depression, for example, in subgenual cingulated gyrus (SCG) DBS, a non-pharmacological treatment for refractory MDD, where a “biphasic effect” has been observed clinically, whereby patients show significant improvement in the first week after electrode implantation, and thereafter the efficacy declines until six months after the procedure when a plateau is reached with slow improvement [134]. The immediate postoperative ameliorative effect is associated with acute inflammation caused by electrodes, accompanied by increased expression of glial fibrillary-acidic-protein, inflammatory mediators (e.g., TNF-α), and p11; the latter plays an important role in the production and utilization of 5-HT [135].

However, an increasing number of studies have suggested that DBS can alleviate chronic inflammation and that the anti-inflammatory effect may be a mechanism for its efficacy. DBS of the lateral cerebellar nucleus in rats inhibited pro-inflammatory gene expression and microglial activation in the surgical group, suggesting that the efficacy of DBS may be related to its anti-inflammatory effects [136]. Stimulation of the anterior thalamic nucleus with DBS for one week resulted in significant decreases in caspase3 activity and interleukin-6 (IL-6) levels in the hippocampus of rats, showing anti-inflammatory and anti-apoptotic effects. However, DBS did not affect TNF-α levels, suggesting that the effect of DBS on cytokines may be specific [137]. The anti-inflammatory effects of DBS may be related to CX3CL1/CX3CR1 signaling. DBS in the subthalamic nucleus reduced the expression of fractalkine (CX3CL1) and its receptor (CX3CR1), inhibited microglial activation and NF-κB expression, reduced pro-inflammatory cytokines IL-1β and IL-6, and increased the expression of the extracellular signal-regulated kinase (ERK) and cleaved-caspase3 [138], suggesting the anti-inflammatory effect caused by DBS treatment.

### Vagus nerve stimulation

VNS is a stimulating electrode placed on the vagus nerve (most often in the neck) that delivers low-frequency, intermittent electrical pulses, approved for refractory depression in 2005 by the FDA. Afferent vagus nerve fibers terminate in the medulla, from which there are projections to many areas of the brain, including the limbic forebrain. VNS affects many brain regions involved in depressive pathology, neurotransmitters (serotonin, norepinephrine), and signal transduction mechanisms (brain-derived neurotrophic factor—tropomyosin receptor kinase B), but the exact mechanism of action is unclear. VNS attenuates the inflammatory response, peripherally (cytokine alterations) and centrally (reduced microglial activation), which is known as the parasympathetic anti-inflammatory pathway [139, 140]. Stress induces sympathetic excitation, increased catecholamine release, and activation of the NF-kB pathway [141] associated with increased brain and peripheral cytokine expression [142]. α- and β-adrenergic receptor activators indirectly activate NF-kB [143], and α-adrenergic receptor blockers reduce stress-induced IL-6 elevation in peripheral blood in humans [144]. Parasympathetic excitation and acetylcholine release activate the A7 subunit of the nicotinic acetylcholine receptor (NAChR), which regulates the transcription and translation of cytokines and exerts anti-inflammatory effects [145–147]. Studies have shown a 42–53.1% two-year remission rate for refractory depression with VNS (≥50% reduction in HDRS scores from baseline) [148, 149]. And a growing number of studies have found that non-invasive VNS (percutaneous or transaural) is as effective as invasive direct stimulation in treating refractory epilepsy [150], Alzheimer’s disease [151], pain, anxiety, depression, etc. [152], predicting the promising application in the treatment on neuropsychiatric disorders.

## Discussion

Neuromodulation therapies that target and disrupt a dysfunctional brain focus, region, or network offer important adjuvant therapies for refractory psychiatric disorders. Advances in our understanding of neuroanatomical networks and the mechanism of stimulation, coupled with developments in material science, miniaturization, energy storage, and delivery, will expand the use of neuromodulation devices in the future. Clinically accessible biomarkers that can indicate the physiological changes precede and after treatment are valuable.

As stated above, mental illness is closely related to the inflammatory response of the body, and inflammation is not only present in the periphery but also affects the central neural circuits that are usually targeted by neuromodulation therapy [153, 154]. Almost all neuromodulation methods have been reported to affect the inflammatory response of the body during application. It is reasonable to speculate that the detection of clinically accessible TSPO-PET and peripheral inflammatory factors could be used as biomarkers.

The use of TSPO as a clinical neuroimaging biomarker of microglia activation and neuroinflammation has increased exponentially in the last decade. Elevated TSPO binding was observed in six of seven studies of unmedicated patients with MDD [155]. Attwells et al. found that elevated prefrontal and anterior cingulate TSPO signal in patients with refractory MDD predicted a reduction in depressive symptoms in patients taking celecoxib (a nonsteroidal anti-inflammatory drug), which indicate that TSPO-PET could predict treatment response [156]. These studies support the implication value of TSPO-PET. However, psychiatric disorders are more heterogeneous in neuropathology and several factors may affect the reproducibility of results and retard the broad use of TSPO-PET at present. These factors include TSPO radioligand used (R-PK11195 or second generation), short-term and long-term outcomes, PET imaging quantification with and without radioligand plasma input data, medicated vs naïve subjects, as well as corrections for the human TSPO polymorphism and vascular radioligand binding [157]. Thus, large sample size studies and standards of operation are needed so that future studies and applications can be appropriate. Fortunately, improved radioligands, especially [11C]ER176, overcome some of the major drawbacks of earlier tracers making the TSPO-PET technique more promising as a neuroinflammatory index.

Numerous studies have provided evidence of increased levels of inflammatory markers in depression and anxiety. Studies have shown that inflammatory factors IL-1β, IL-6, TNF-α, and CRP are elevated and anti-inflammatory factors IL-4, IL-8, and IL-10 are decreased in peripheral and cerebrospinal fluid and correlate with patients’ symptoms and disease duration in depression and anxiety [158–160]. Remission of depressive symptoms accompanied by a decrease in inflammatory factors [161]. Available data suggest that multiple kinds of antidepressants significantly downregulate a wide array of peripheral biomarkers such as IL-1ß, IL-2, IL-5, IL-6, IL-8, IL-10, CRP, or IFN-γ and inhibit inflammation in the brain [162].

For the question of whether peripheral cytokines reflect the inflammatory conditions in CNS, there are channels for peripheral inflammatory factors to interact with central inflammation:(1) cross the blood-brain barrier through leakage zones, (2) active transport of supersaturated transport molecules, (3) activation of cerebrovascular endothelial cells and other cell types (including perivascular macrophages, which then produce cytokines and other inflammatory mediators in the brain), and (4) bind to cytokine receptors with peripheral afferent nerve fibers (e.g., vagus nerve) [163]. (5) propose inflammatory vesicle activation and immune cell transport [164, 165]. Studies also showed a positive relation between peripheral inflammatory factors and central inflammation in depression. For example, in a sample that examined three cohorts (two of whom were experiencing MDD), peripheral inflammation factors (prostaglandin E2/CRP) and (TNFα/CRP) consistently correlated with brain TSPO signal and had sufficient positive predictive value to be considered for use in clinical trials [166]. Not all patients with mood disorders have elevated levels of inflammation, but in patients with a high inflammatory background, pro-inflammatory factors are suggestive of the condition and may benefit future trials exploring anti-inflammatory treatment options for anxiety and depression.

There are limitations that remain. The activation of neuroinflammation is not consistently reported by all patients and appears not to be specific to any particular category. We need to understand the underlying physiology of microglial activation. In addition, neuromodulation has shown promising effects in reducing cytokines and improving microglial polarization; however, the exact mechanism is unclear and needs to be further explored. Furthermore, some studies have shown that neuromodulation techniques play a role in astrocyte-associated inflammatory responses, which may lead to confusion. Notably, the neuromodulation technique did not show these effects in healthy individuals, suggesting that it may reduce an abnormal excess of inflammatory and pro-inflammatory factors and increase anti-inflammatory factors to promote the restoration of homeostasis when the inflammatory balance is disrupted [167, 168]. The key challenges to effective treatment are defining the targets for stimulation and multiple configurable stimulation measures, including stimulation pathways, frequency, pulse width, duration, intensity, and stimulation duration. The application of neuroinflammatory biomarkers in neuromodulation therapies will inevitably rely on more clinical evidence and data science to achieve the best outcomes, particularly chronically obtained data.

## Conclusion

Inflammatory biomarkers may serve as a reference for the evaluation of anxiety and depression and treatment selection. Neuromodulation therapy targeting neural circuit dysfunction to treat affective disorders by reducing neuroinflammation provides a better understanding of the pathogenesis of the disease and objectively evaluates the efficacy of physical therapy.
